# Utilizing statistical analysis for motion imagination classification in brain-computer interface systems

**DOI:** 10.1371/journal.pone.0327121

**Published:** 2025-07-08

**Authors:** Yang Li, Jingyu Zhang

**Affiliations:** College of Physical Education, Changchun Normal University, Changchun, Ji Lin, China; Universidad de Almeria, SPAIN

## Abstract

In this study, we introduce a novel Field-Agnostic Riemannian-Kernel Alignment (FARKA) method to advance the classification of motion imagination in Brain-Computer Interface (BCI) systems. BCI systems enable direct control of external devices through brain activity, bypassing peripheral nerves and muscles. Among various BCI technologies, electroencephalography (EEG) based on non-intrusive cortical potential signals stands out due to its high temporal resolution and non-invasive nature. EEG-based BCI technology encodes human brain intentions into cortical potentials, which are recorded and decoded into control commands. This technology is crucial for applications in motion rehabilitation, training optimization, and motion control. The proposed FARKA method combines Riemannian Alignment for sample alignment, Riemannian Tangent Space for spatial representation extraction, and Knowledge Kernel Adaptation to learn field-agnostic kernel matrices. Our approach addresses the limitations of current methods by enhancing classification performance and efficiency in inter-individual MI tasks. Experimental results on three public EEG datasets demonstrate the superior performance of FARKA compared to existing methods.

## 1. Introduction

As a novel communication system in Human-Computer Interaction (HCI), Brain-Computer Interface (BCI) enables direct control of external devices through brain activity, bypassing peripheral nerves and muscles, thus establishing a new bridge between the human brain and devices [1,2]. Among various BCI technologies, electroencephalography (EEG) based on non-intrusive cortical potential signals stands out due to its high temporal resolution and the absence of ethical and intrusive surgical constraints [3]. It has become a key technology for developing BCI systems that involve typical users. Specifically, EEG-based BCI technology encodes human brain intentions into cortical potentials, which are then recorded and decoded into control commands by devices. Through real-time monitoring and feedback of brain activity, it plays a crucial role in motion rehabilitation, training optimization, motion control, and research [4].

EEG-based BCI technology involves interdisciplinary integration of signal processing, machine learning, and cognitive neuroscience. Among the various EEG-based BCI techniques, three mainstream paradigms are event-related potentials (ERPs), steady-state visual evoked potentials (SSVEPs), and sensorimotor rhythms (SMRs) [5]. Notably, voluntary motion imagination (MI) induces sensorimotor rhythms autonomously without additional stimuli. It can effectively enhance physical training by improving motion skills, boosting psychological readiness, and optimizing training outcomes. The MI process in typical individuals results in event-related desynchronization (ERD) of mu and beta rhythms in EEG signals: for instance, MI of the left limb leads to energy suppression in the corresponding rhythm in the right hemisphere of the brain, and vice versa. This ERD phenomenon is embedded in the nonlinear, non-stationary, and low signal-to-noise ratio EEG signals, and is subject to interference from temporal and spatial coupling characteristics, making the classification of different MI types challenging [6].

EEG signals, as typical multidimensional time series, have spatial representations that can stably express ERD phenomena related to different MI types, which are often used for signal decoding. Common spatial representations include Riemannian Tangent Space (RTS) [7] and Common Spatial Patterns (CSP) [8]. CSP representations are typically used for binary classification, aiming to maximize the variance of one MI category while minimizing the variance of another through optimal spatial representation transformation. RTS representations project EEG samples into Riemannian geometric space and generate representation vectors at the tangent points in Euclidean space. To achieve classification of EEG-based MI, classifiers such as Linear Discriminant Analysis [9] or Kernel Support Vector Machines (KSVM) [10] are often employed to classify CSP and RTS spatial representations.

However, due to the diverse user population of MI-based BCI systems, varying states of individuals during EEG sample collection, and differences in collection device individuals, EEG samples exhibit diverse distributions across individuals, which violate the assumption of independent and identically distributed (i.i.d.) samples in machine learning. Consequently, it is difficult to construct classifiers based on representation sets across individuals for motion imagination signal classification. To address inter-individual classification, knowledge adaptation [11] methods are utilized. Specifically, these methods treat each individual’s sample set as an single field and implement inter-field classification approaches, including sample alignment methods, representation adaptation methods, and deep learning methods.

Sample alignment methods for inter-individual motion imagination classification originate from field adaptation, which aligns different individuals’ sample sets to the same or similar distributions [12]. Since statistical information for EEG is embedded in the covariance matrix, researchers use the centroid of the covariance matrix for sample alignment. The Riemannian Alignment (RA) [13] method first aligns the covariance matrix centroids of EEG samples from different individuals to the identity matrix, successfully reducing sample distribution differences, and employs Minimum Distance Mean (MDM) [14] classifier for direct classification of the aligned covariance matrices. However, due to the high computational complexity of centroids in Riemannian space, Euclidean Alignment (EA) [15] calculates covariance matrix centroids in Euclidean space and efficiently aligns samples to the identity matrix. The aligned samples can then extract CSP or RTS representations for more flexible classification. Recently, based on RA and EA methods, researchers first extract CSP representations in sub-bands and then implement target alignment for spatial representation alignment, significantly improving inter-individual motion imagination classification accuracy.

Representation adaptation methods for inter-individual motion imagination classification stem from field adaptation, which divides spatial representation sets (usually CSP or RTS) into source and target fields and adapts between the two fields. The earliest field adaptation method, i.e., Transfer Component Analysis (TCA) [16], matches marginal probability distributions of source and target fields. To enhance sample distinguishability in the target field, researchers proposed Joint Distribution Alignment [17], Dynamic Distribution Alignment [18], and Joint Probability Distribution Alignment [19] methods based on pseudo-labels of target field samples, aligning both marginal and conditional probability distributions. To improve representations during field adaptation, Transfer Joint Matching (TJM) method [20] selects sparse representations using l2,1 norm, reducing the interference of redundant representations in the adaptation process. Building on this, researchers proposed Manifold Embedded Knowledge Transfer [21] and Manifold Embedded Transfer Learning [22] methods based on subspace field adaptation with additional constraints, further developing more generalized inter-individual MI classifiers based on CSP or RTS representations. Recently, combining weighted regularized CSP representations with JPDA method has constructed a higher-performance and efficient inter-individual motion imagination classification approach. Additionally, building on [21] and [22], researchers developed Multi-Manifold Embedded Distribution Alignment method [23], which enhances inter-individual classification performance by maximizing intra-class distances and minimizing inter-class distances.

Deep learning methods for inter-individual motion imagination classification are based on DANN [24], leveraging the end-to-end characteristics of deep learning models to automatically learn field-agnostic representations. Recently, deep field adaptation representation models have achieved end-to-end representation extraction, field discrimination, and classification optimization, obtaining field-agnostic deep representations for EEG samples. To address sub-field adaptation issues of different MI categories, the Dynamic Joint DANN model [25] proposed using multiple sub-field discriminators to improves the field adversarial learning process, significantly enhancing classification accuracy. Furthermore, researchers introduced Wasserstein distance to measure representations learned by deep field adaptation representation models, ensuring separability of different MI categories in the target field and further improving inter-individual motion imagination classification performance [26].

Clearly, the aim of inter-individual motion imagination classification is to obtain field-agnostic representations to address the problem of sample sets between populations not conforming to i.i.d., thereby hindering generalization. Formally, solutions to this problem include two branches: one is field adaptation methods based on CSP or RTS representations, which fundamentally aim to minimize MMD through representation transformation [27]; the other is field adversarial methods based on DANN models, which obtain field-agnostic deep representations through the learnable characteristics of neural networks [28]. However, both branches have limitations and are not directly applicable to the construction of real-time online MI-based BCI systems. The former is constrained by the expression of sample distribution differences, and MMD-based representation transformation does not fully characterize representation distributions, especially for nonlinear and non-stationary EEG samples. The latter, while capable of learning field-agnostic representations and handling the nonlinear and non-stationary nature of EEG for MI, suffers from long training times and unstable convergence of the minimax optimization, severely limiting its practical application.

To address the current bottlenecks in inter-individual motion imagination classification, we propose a novel Field-Agnostic Riemannian-Kernel Alignment (FARKA) method. This method first employs RA to align field sample sets, then extracts Riemannian tangent space representations, and finally uses the knowledge kernel adaptation method to learn field-agnostic kernel matrices and extract field-agnostic representations using these matrices. The proposed FARKA bypasses the minimization of maximum mean discrepancy, instead learning field-agnostic representations through field-agnostic kernel matrices, offering a more efficient field-agnostic representation learning process compared to the DANN model. Moreover, the knowledge kernel adaptation method has been successfully proven to handle the nonlinear and non-stationary characteristics of EEG for MI when applied to common spatial pattern representations. In summary, our FARKA method have the following key contributions:

(1) It integrates the completeness of Riemannian tangent space representations with the field-agnostic representation capability of knowledge kernel adaptation, overcoming the issues of incomplete characterization of EEG for MI by maximum mean discrepancy minimization and the high time complexity and convergence difficulties of the DANN model, further enhancing inter-individual motion imagination classification performance.
(2) It mitigates the dimensionality disaster in the representation filed adaptation process, offering good execution efficiency and wide applicability to real-time online MI-based BCI systems. Furthermore, its feasibility and effectiveness have been validated on three public datasets using two common inter-individual strategies.

## 2. Related works

Motion imagination is a common application of non-intrusive BCI systems [29]. It involves imagining the movement of one’s limbs or muscles without actual physical movement. This process is fundamentally a cognitive perception, representing a psychological state without any peripheral muscle activity. During motion imagination, specific brain regions become consciously activated, and these regions have been confirmed to participate in the preparation and execution of actual bodily movements. Motion imagination has significant potential in the field of sports. It can effectively enhance athletes’ performance and skills while aiding in the improvement of motion coordination and accuracy during training. By simulating the process of actual movement, motion imagination helps athletes practice skills and adjust tactics without physical exertion. Especially in high-level competitive sports, motion imagination serves as an effective tool for boosting training effectiveness, enhancing athletic ability, and improving mental preparation.

During motion imagination, the brain generates EEG signals, primarily containing μrhythms (8–13 Hz) and β rhythms (13–30 Hz). The processing of motion imagination EEG signals involves three main stages: preprocessing, representation extraction, and representation classification. EEG signals are characterized by their low amplitude, non-stationarity, and non-linearity, which necessitates specialized equipment for collection. Despite this, noise artifacts may still be present. The preprocessing stage aims to reduce noise and improve the signal-to-noise ratio while extracting the required frequency bands based on experimental needs. The representation extraction stage decodes the preprocessed EEG signals to extract representations that represent motion imagination. The representation classification stage constructs a suitable classifier to categorize the extracted EEG representations and produce classification results.

### 2.1 Common preprocessing methods

In the experimental process, EEG signals for MI are induced by stimuli. The EEG signals are collected and amplified via an electrode cap and then transmitted to a computer as raw signals. Due to noise from the environment and physiological factors such as electrooculographic, electromyographic, and electrocardiographic interference, the raw EEG signals contain noise artifacts that can significantly degrade system performance. Preprocessing algorithms can effectively reduce noise components in EEG signals, improving the signal-to-noise ratio and yielding accurate EEG data. This section will introduce common preprocessing methods in the field of motion imagination EEG signal classification.

#### 2.1.1 Wavelet transform algorithm.

The wavelet transform algorithm builds upon the Fourier transform [30]. The Fourier transform is a standard tool in signal analysis that decomposes a signal into its frequency components and determines their relative strengths. The Fourier transform and its inverse are given by:


$$F(\omega )=\int_{-\infty }^{\infty }f(t)e^{-j\omega t}dt$$ (1)

$$f(t)=\frac{1}{2\pi }\int_{-\infty }^{\infty }F(\omega )e^{j\omega t}d\omega$$ (2)

This transform is primarily applied to stationary signals, where characteristics do not vary over time. For non-stationary signals, the Short-Time Fourier Transform (STFT) is used, introducing a local frequency parameter with a “window” approach. The STFT is defined as:


$$F\mathrm{\backslash nolimits}(\omega ,\tau )=\int_{-\infty }^{\infty }f(t)\psi^{*}(t-\tau )e^{-j\omega t}dt$$ (3)

where *ψ*(*t*) is the window function. The STFT uses the window function *ψ*(*t*) to analyze signals in segments, obtaining time information of frequency components and constructing local spectral representations. However, since EEG signals are non-stationary with time-varying distribution parameters, a fixed window function may not meet their frequency requirements. The wavelet transform builds upon the STFT’s localized approach, using wavelet bases to overcome the limitation of a fixed window function [31]. It enables both time and frequency domain localization, processing low and high-frequency components simultaneously and addressing the shortcomings of the Fourier transform. The basic expression of the wavelet transform is:


$$W_{\psi }f(a,b)=\frac{1}{\sqrt{\left|a\right|}}\int_{-\infty }^{\infty }f(t)\psi^{*}\left(\frac{t-b}{a}\right)dt=\left\langle f,\psi_{a,b}\right\rangle$$ (4)

where *a* is the scaling factor representing the contraction of the wavelet basis, *b* is the translation factor representing the shift, and *ψ*_*a,b*_ is the wavelet basis function, also known as the mother wavelet [32]. The wavelet transform replaces the infinite-length triangular function basis in STFT with wavelet bases, which can be scaled and shifted according to the signal, providing a time-frequency window that changes with frequency and achieving multi-scale detail refinement [33].

#### 2.1.2 Independent component analysis.

Independent Component Analysis (ICA) is a signal processing and data analysis algorithm used to decompose multivariate signals into independent, non-Gaussian components [34]. The basic idea is to assume that a signal is a linear combination of unknown independent sources and then estimate these sources based on the observed signals. The fundamental principle of ICA is:

Assume *X* is a matrix composed of *n* linearly mixed signals *x*_1_, *x*_2_,..., *x*_*n*_, and *S* is a matrix composed of *n* independent components *s*_1_, *s*_2_,..., *s*_*n*_. *A* is an n*m mixing matrix, such that:


X=AS (5)

This model is known as the ICA model. ICA is a generative model that represents how mixed signals are generated through independent components *s*_*i*_. The independent components are latent variables that cannot be directly observed, and the mixing matrix *A* is also unknown. The goal of ICA is to find a m*n separation matrix *W* such that:


S=WX (6)

From this equation, the independent components *S* can be obtained.

ICA separates mixed signal components to extract relatively independent signal sources, which have higher information entropy and are significant for signal analysis, representation extraction, signal processing, and pattern recognition applications. In practical applications, ICA is widely used in signal processing, image processing, speech analysis, financial data analysis, and more. In EEG signal processing, ICA can isolate activities from different neural sources, which is useful for identifying specific brain activities and exploring interactions between brain regions. It is also used to remove artifacts from electrooculographic, electrocardiographic, and electromyographic interferences.

### 2.2 Common representation extraction algorithms

In BCI systems, representation extraction is a crucial step. Although preprocessing reduces some noise in the EEG signals, the data still have high dimensionality. Representation extraction analyzes the temporal, spectral, and time-frequency characteristics of EEG signals, converting the raw signals into a set of values or representation vectors that represent the signal characteristics. This process reduces dimensionality, decreases computational complexity, and shortens processing time for subsequent classification.

Common representation extraction algorithms can be categorized as follows:

(1) Time-domain Algorithms: These include variance, slope, and mean amplitude. These algorithms are simple and fast but perform poorly with nonlinear or non-stationary signals [35].
(2) Frequency-domain Algorithms: These include power spectral density estimation and coherence analysis. While these algorithms can extract spectral features of signals, they suffer from varying degrees of time-frequency blurring [36].
(3) Time-frequency Domain Algorithms: Examples include wavelet transform and empirical mode decomposition (EMD) [37]. These algorithms avoid the time-frequency blurring issues present in frequency-domain algorithms but have higher computational complexity.
(4) Nonlinear Algorithms: Examples include Hurst Exponent (HE) [38], Approximate Entropy (ApEn) [39], and Fuzzy Entropy (FuzzyEn) [40]. These algorithms reflect the nonlinear and non-stationary characteristics of EEG signals but are computationally intensive and require careful selection and combination of representations [41].

This section introduces some common methods for processing EEG signal representations.

#### 2.2.1 Empirical mode decomposition.

EMD is a signal processing algorithm based on local signal characteristics [42]. It decomposes complex nonlinear and non-stationary signals into a sum of intrinsic mode functions (IMFs) [43]. The basic principle involves representing a signal as a sum of oscillations of different frequencies, where low-frequency oscillations correspond to large time-scale changes and high-frequency oscillations to small time-scale changes.

In EEG signal analysis, EMD can extract ERPs and oscillatory components. ERPs are waveforms related to specific events observed in EEG signals, such as brain responses to auditory or visual stimuli. Oscillatory components refer to steady-state waveforms appearing in various frequency ranges. For a signal *x*(*t*), the basic steps of EMD are:

1) Extract the maxima and minima of *x*(*t*) and fit curves using linear or cubic spline interpolation to obtain the upper and lower envelopes, denoted as *e*_*max*_(*t*) and *e*_*min*_(*t*).
2) Compute the mean of the upper and lower envelopes:

$$s_{1}(t)=\frac{e_{\max}(t)+e_{\min}(t)}{2}$$ (7)

3) Subtract the mean envelope from the original signal to obtain the initial mode function:

$$c_{1,1}(t)=x(t)-s_{1}(t)$$ (8)

4) Check if *c*_1,1_(*t*) is a single-frequency IMF. If not, repeat steps 1–3 with *c*_1,1_(*t*) as the new signal until *c*_1,*k*_(*t*) is a single-frequency IMF, which becomes the first IMF component *I*_1_(*t*):

$$c_{1,k}(t)=c_{1,k-1}(t)-s_{k}(t)$$ (9)

$$I_{1}(t)=c_{1,k}(t)$$ (10)

5) Subtract *I*_1_(*t*) from the original signal to obtain the residual *r*_1_(*t*) and use it to repeat the process until *r*_*n*_(*t*) is a monotonic function or has only one extremum.

$$r_{1}(t)=x(t)-I_{1}(t)$$ (11)

6) The residual *r*_*n*_(*t*) and all IMFs sum up to the original signal:

$$x(t)=\sum_{i=1}^{n}I_{i}(t)+r_{n}(t)$$ (12)

#### 2.2.2 Common spatial pattern.

The CSP algorithm is a spatial filtering representation extraction method. Its core idea is to project the EEG signals onto a new spatial domain using a weighted sum approach, maximizing the ability to distinguish between different motion imagination tasks [44]. CSP is known for its stability and robustness and is widely used in EEG signal processing across various frequency bands and time windows. The algorithm steps are:

1) Represent the EEG signals in matrix form *X*(*N* × *T*), where *N* is the number of EEG channels and *T* is the number of signal points per channel. Classify the raw EEG data into two classes, *E*_1_ and *E*_2_, for left and right hand motion imagination tasks, respectively.
2) Compute the covariance matrices for the two classes:

$$R_{i}=\frac{E_{i}E_{i}^{T}}{trace(E_{i}E_{i}^{T})}$$ (13)

3) Compute the mixed-space covariance matrix:

$$R={\bar{R}}_{1}+{\bar{R}}_{2}$$ (14)

4) Perform eigenvalue decomposition on the mixed-space covariance matrix *R*:

$$R=U\lambda U^{T}$$ (15)

where *U* is the matrix of eigenvectors andλ is the diagonal matrix of eigenvalues.

5) Arrange the eigenvalues in descending order and compute the whitening matrix:

$$P=\sqrt{\lambda^{-1}}U^{T}$$ (16)

6) Construct spatial filters:

$$S_{1}=PR_{1}P^{T},S_{2}=PR_{2}P^{T}$$ (17)

Perform Principal Component Analysis (PCA) on *S*_1_ and *S*_2_ to obtain:


$$S_{1}=B_{1}\lambda_{1}B_{1}^{T},S_{2}=B_{2}\lambda_{2}B_{2}^{T}$$ (18)

where *B*_1_ = *B*_2_ = *V* and the sum of $\lambda_{1}$ and $\lambda_{2}$ equals the identity matrix. When the eigenvalue of *S*_1_ is maximized, the eigenvalue of *S*_2_ is minimized, and vice versa. The optimal spatial filter projection matrix is:


$$W=B^{T}P$$ (19)

7) Project the EEG data using the projection matrix to obtain the representation matrix:

$$Z_{N\times T}=W_{N\times T}E_{N\times T}$$ (20)

Hereafter, select the top *m* and bottom *m* rows of matrix *Z* (where 2*m* < *N*) as EEG representations.

#### 2.2.3 Power spectral density.

Power Spectral Density (PSD) [45] is a commonly used analysis algorithm in signal processing that describes the distribution of signal energy across different frequencies. In EEG signal analysis, PSD is widely used to analyze frequency domain representations, including frequency distribution, energy density, and band characteristics [46]. Common PSD algorithms include periodogram, windowed averaging periodogram, and autoregressive model (AR).

The periodogram method involves applying Fourier transform directly to the EEG signal and squaring the magnitude of the result to obtain the spectral values:


$$\hat{P}(\omega )=\frac{1}{N}|X_{N}(e^{-j\omega }){|}^{2}=\frac{1}{N}|\sum_{n=0}^{N-1}x(n)e^{-j\omega n}{|}^{2}$$ (21)

The windowed averaging periodogram method averages multiple periodograms to reduce variance, dividing the signal into segments based on selected window length and overlap, and applying a window function (e.g., Hanning or Hamming). The Fourier transform is then applied to each segment, and the power spectra are averaged to estimate the final PSD. This method mitigates issues such as low spectral resolution and spectral leakage but still requires careful selection of segment number and window length.

The autoregressive model method predicts future observations as a linear combination of past observations and white noise [47]:


$$x(n)=\sum_{i=1}^{m}a_{i}x(n-i)+u(n)$$ (22)

where *x*(*n*) is the signal sequence, *u*(*n*) is white noise, and *a*_*i*_ are model parameters. The power spectral density can be derived from the Z-transform:


$$H(z)=x(z)/U(z)=1/\left(1+\sum_{i=1}^{m}a_{i}z^{-i}\right)=X(z)/\sigma$$ (23)

The power spectral density can be easily obtained from (23) and Fourier transform as follows:


$$\hat{P}(\omega )=X(e^{i\omega }{)}^{2}=\sigma^{2}H(e^{j\omega }{)}^{2}=\frac{\sigma^{2}}{|1+\sum_{k=1}^{m}a_{k}e^{-j\omega k}{|}^{2}}$$ (24)

The AR model effectively describes autocorrelations in time series data and is simple and intuitive, making it suitable for stable time series data prediction.

#### 2.2.4 Sample entropy.

Sample Entropy (SampEn) is a parameter introduced by [48] to characterize the complexity of time series. It extends the concept of Approximate Entropy, maintaining its ability to describe time series complexity while being less influenced by data length, more robust against noise, and showing better consistency compared to other methods. The calculation process for Sample Entropy is:

1) Given a time series of length *N*, calculate the sample entropy:

X={x(1),x(2),…,x(N)} (25)

2) Construct *m*-dimensional vectors from the time series:

X(i)={x(i),x(i+1),⋯,x(i+m−1)} (26)

where *i*=1,2,…,*N*−*m*+1.

3) Define the distance between two vectors *X*(*i*) and *X*(*j*) as:

$$d[X(i),X(j)]={\text{max}}_{k\in (0,m-1)}|x(i+k)-x(j+k)|$$ (27)

where *i* ≠ *j*, it is the largest difference between the two corresponding elements.

4) Given a threshold *r* (*r* > 0), count the number of pairs where *d*[*X*(*i*),*X*(*j*)]<*r*, and calculate:

$$B_{i}^{m}(r)=\frac{1}{N-m}num\{d[X(i),X(j)]<r\}$$ (28)

Average this for all results to obtain:


$$B^{m}(r)=\frac{1}{N-m+1}\sum_{i=1}^{N-m+1}B_{i}^{m}(r)$$ (29)

5) Increase the dimension *m* by 1, repeat steps 1–3 to compute *B*_*m*+1_(*r*), and derive the theoretical sample entropy as:

$$SampEn\mathrm{\backslash nolimits}(m,r)=\underset{N\to \infty }{\lim}\{-\ln[\frac{B^{m+1}(r)}{B^{m}(r)}\}$$ (30)

For finite *N*, the estimated sample entropy is:


$$SampEn\mathrm{\backslash nolimits}(m,r,N)=-\ln[\frac{B^{m+1}(r)}{B^{m}(r)}]$$ (31)

### 2.3 Common motion imagination representation classification algorithms

After preprocessing and representation extraction, EEG signals are transformed into lower-dimensional representations. By applying representation classification models, a BCI system can identify motion imagination classes, enabling control of associated actions. This is crucial for rehabilitation training for motion-impaired patients and for BCI control in healthy individuals. This section introduces some common classification algorithms for motion imagination EEG signal representations.

#### 2.3.1 Support vector machine.

Support Vector Machines (SVM) are a type of supervised learning model used for binary classification, commonly employed in classification and regression analysis [49]. The core idea is to solve a convex quadratic optimization problem to find an optimal hyperplane in the representation space that separates different classes of data points, while maximizing the distance between the hyperplane and the nearest data points, thereby improving the model’s classification accuracy and generalization capability [50]. For binary classification, the hyperplane can be described as:


$$\omega^{T\mathrm{\backslash nolimits}}x+b=0$$ (32)

where *ω* is the normal vector of the hyperplane, representing its direction, and *b* is the displacement term, representing the distance from the hyperplane to the origin. The distance *r* of any point in the sample space to the hyperplane is:


$$r=\frac{\left|\omega^{T}x+b\right|}{\left\|\omega \right\|}$$ (33)

The sum of the distances of the support vectors from the hyperplane is *ω*/2*.* To maximize this margin, which involves solving a convex quadratic problem, it is evident that we need to maximize ||*ω*||^−1^, which is equivalent to minimizing ||*ω*||^2^. Thus, the basic form of the SVM is:


$${\text{min}}_{\omega ,b}\frac{1}{2}{\left\|\omega \right\|}^{2}s\mathrm{\backslash nolimits}.t.y(\omega^{T}x_{i}+b)\geq 1,i=1,2,\ldots ,n$$ (34)

When some training samples cannot satisfy the condition *y*_*i*_(*ω*^*T*^*x*_*i*_ + *b*)≥1, i.e., when the data is linearly inseparable, the geometric margin, being a distance, is non-negative, and noisy data can render the problem unsolvable. In such cases, slack variables ξi ≥ 0 are introduced to allow some points to be within the margin:


$$y_{i}(\omega^{T}x_{i}+b)\geq 1-\varepsilon_{i},i=1,2,\ldots ,n$$ (35)

In practice, data often need to be analyzed in a nonlinear context, which may not be separable in the original representation space. Kernel functions are used to map the data into a higher-dimensional representation space where it becomes separable. The choice of kernel function directly affects the classification performance of the SVM. Common kernel functions include:

1) Linear Kernel:

$$\kappa (x_{i},x_{j})=x_{i}^{T}x_{j}$$ (36)

2) Polynomial Kernel:

$$\kappa (xi,x_{j})=(x_{i}^{T}x_{j}{)}^{d}$$ (37)

3) Gaussian (RBF) Kernel:

$$\kappa (x_{i},x_{j})=\exp\left(-\frac{{\left\|x_{i}-x_{j}\right\|}^{2}}{2\sigma^{2}}\right)$$ (38)

4) Laplacian Kernel:

$$\kappa (x_{i},x_{j})=\exp(-\frac{\left\|x_{i}-x_{j}\right\|}{\sigma })$$ (39)

5) Sigmoid Kernel:

$$\kappa (x_{i},x_{j})=\tanh(\beta x_{i}^{T}x_{j}+\theta )$$ (40)

SVMs can handle high-dimensional representation spaces and small sample sizes, making them well-suited for motion imagination classification tasks due to their robust classification performance and generalization capability.

#### 2.3.2 k-nearest neighbors.

The k-Nearest Neighbors (KNN) algorithm is a common supervised learning method used for classification and regression problems [51]. Its basic principle is to identify the *k* most similar samples to the query sample and then determine the class of the query sample based on the statistical analysis of these *k* neighbors. KNN is a simple and easy-to-understand algorithm that is applicable to multi-class problems and does not require training. However, it requires calculating the distance between the test sample and all training samples, which can be computationally intensive, and it is sensitive to the distances of neighboring points. Consequently, its classification performance may be affected by noisy datasets [52].

The basic steps of KNN are as follows: Calculate the distance between the query sample and each known sample using distance metrics such as Euclidean distance, Manhattan distance, or cosine similarity. Select the *k* nearest known samples, often using a weighted average method, and vote on the class of these *k* samples to determine the class of the query sample. If *k* is not appropriately chosen, overfitting or underfitting may occur, so cross-validation is often used to determine the optimal value of *k*.

#### 2.3.3 Convolutional neural networks.

Convolutional Neural Networks (ConvNets) are deep neural networks with convolutional structures. ConvNets are effective at representation extraction and optimization in high-dimensional representation spaces. A typical ConvNet consists of convolutional layers, pooling layers, and fully connected layers [53].

The convolutional layer is primarily responsible for representation extraction. Convolution operations scan input data using convolutional kernels to produce feature maps and capture local representations. This layer contains multiple convolutional kernels, and the weights and biases of these kernels are optimized through training. The convolution operation is given by:


$${Y\mathrm{\backslash nolimits}}_{n}=\sum_{i=1}^{M\mathrm{\backslash nolimits}}\left[({W\mathrm{\backslash nolimits}}_{n}^{i}*x_{i})+b_{n}\right]$$ (41)

where *x*_*i*_ represents the input representations, ${W\mathrm{\backslash nolimits}}_{n}^{i}$ represents the weights, *b*_*n*_ represents the bias, and Y_n_ represents the output representations. The parameters ${W\mathrm{\backslash nolimits}}_{n}^{i}$ and *b*_*n*_ are core parameters of the convolutional layer and are trained using backpropagation to minimize the network’s loss function.

The pooling layer performs down-sampling on the input data to reduce the number of parameters and computational complexity while improving model robustness and generalization. The pooling layer divides the feature maps from the convolutional layer into several local slices and uses a pooling function to compute statistical features for each slice. Common pooling methods include max pooling and average pooling. Max pooling selects the maximum value from each pooling region as the output, while average pooling computes the average value. Therefore, the pooling layer is also known as the down-sampling layer.

The fully connected layer’s main function is to flatten and connect the outputs from the previous convolutional and pooling layers, and then input them into a fully connected layer for classification or regression tasks. It does not perform feature extraction but integrates and consolidates features extracted by previous layers, transforming the feature maps into vectors and losing the spatial topology. In ConvNets, fully connected layers typically follow convolutional and pooling layers, with each neuron connected to all neurons in the previous layer, converting feature maps into output class probabilities.

Traditional ConvNets are primarily used in computer vision and exhibit outstanding performance in image recognition and object detection. Due to the difficulty in collecting EEG signals and the variability among subjects, high-quality data are limited. Traditional ConvNets may suffer from inadequate training and poor classification performance with small sample sizes. To achieve optimal results with limited training samples, ConvNet structures need to be simplified, reducing the number of trainable parameters to ensure that the remaining parameters are adequately trained.

## 3. Materials and methods

### 3.1 Overview

**Fig 1:**
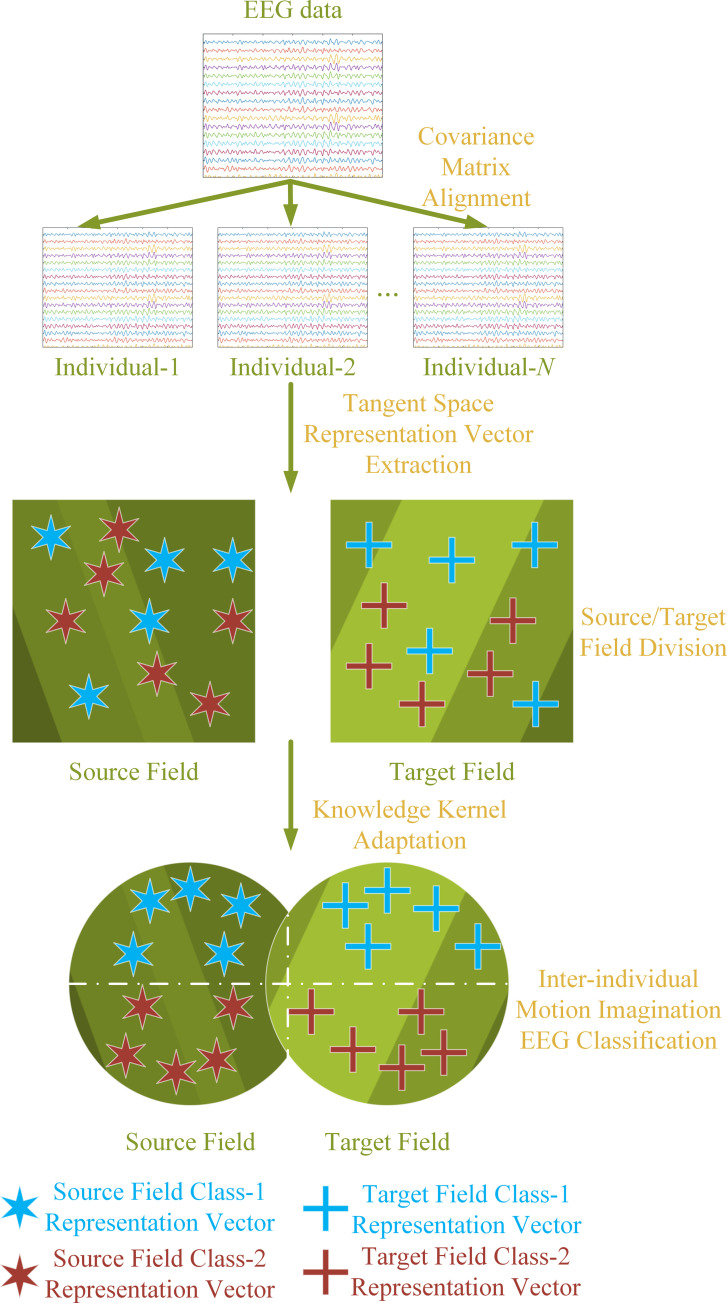
Overall architecture of the proposed Field-Agnostic Riemannian-Kernel Alignment (FARKA) method. This schematic illustrates the key processing stages of the FARKA framework for inter-individual motion imagery EEG classification. It highlights the sequential application of: (1) Sample Covariance Matrix Alignment (e.g., using Riemannian Alignment), (2) Tangent Space Mapping for feature extraction, and (3) Knowledge Kernel Adaptation (KKA) for learning a field-agnostic kernel matrix to enable classification across source and target domains. Arrows indicate the flow of data and processing steps.

As discussed earlier, there is considerable individual variability in EEG signals for MI. To develop a generalizable BCI system across different subjects, it is necessary to design and implement a inter-individual EEG signal representation learning method. Fig 1 illustrates the overall framework of the proposed method, which includes the following steps:

1) Aligning Sample Covariance Matrices: Using the centroid of covariance matrices in Riemannian space as the distribution evaluation criterion, align the sample covariance matrices of different individuals.
2) Tangent Space Mapping: After alignment, perform tangent space mapping on the sample covariance matrices and compute the tangent space representation vectors in the Euclidean space.
3) Source and Target Field Identification: Determine the source and target fields for inter-individual EEG analysis and construct a knowledge kernel adaptation method between these fields.

### 3.2 Sample alignment and spatial representation

To ensure that classifiers trained in the source field generalize well in the target field, it is essential to minimize the distribution differences between fields. EEG data, as multidimensional time series, have covariance matrices with symmetric, positive-definite properties, lying in the symmetric positive definite (SPD) space. The SPD property of covariance matrices allows for various distance metrics, such as Euclidean distance and Riemannian distance, and enables the use of centroids to measure sample distribution. In inter-individual EEG analysis for MI, researchers have employed centroid alignment methods in Riemannian space to align each individual’s sample covariance matrix to the identity matrix, thereby reducing the distribution differences across individuals [54] [55]. This method has become a standard preprocessing step for inter-individual EEG analysis.

In this study, we adopt this alignment method for sample sets from different individuals. Specifically, for two SPD matrices *M*_1_ and *M*_2_, the Riemannian distance can be defined as:


$$\delta (M_{1},M_{2})={\left\|\log(M_{1}^{-1}M_{2})\right\|}_{F}$$ (42)

where ||.|| denotes the Frobenius norm. Based on the Riemannian distance, the Riemannian mean of *n* SPD matrices can be computed as:


$$N_{R}=\underset{M}{\mathrm{\backslash arg}\min}\sum {\mathrm{\backslash nolimits}}_{i=1}^{n}\delta^{2}(M,M_{i})$$ (43)

Given that the covariance matrices of EEG samples for MI are SPD matrices, the centroid in Riemannian space can be calculated using the above formula. For a covariance matrix $M_{c,i}=x_{c,i}x_{c,i}^{T}$ from a individual *c*, alignment is performed using the inverse square root of the Riemannian centroid:


$$Q_{c,i}=N_{R}^{-1/2}M_{c,i}N_{R}^{-1/2}$$ (44)

After alignment, the centroid of individual *c*’s covariance matrix in Riemannian space is:


$${\bar{N}}_{R}=\underset{Q}{\mathrm{\backslash arg}\min}\sum {\mathrm{\backslash nolimits}}_{i=1}^{n}\delta^{2}(Q,Q_{i})=N_{R}^{-1/2}N_{R}N_{R}^{-1/2}=I$$ (45)

Thus, after applying the alignment operation to the covariance matrices of all *C* individuals, all sample covariances can be aligned to the identity matrix *I*, reducing the distribution differences between EEG sample sets. Similarly, due to the properties of SPD matrices, alignment can also be performed in Euclidean space or log-Euclidean space:


$$\left\{ \begin{array}{c} N_{E}=\frac{1}{n}\sum {\mathrm{\backslash nolimits}}_{i=1}^{n}x_{c,i}x_{c,i}^{T}\\ N_{L}=\exp\left(\sum {\mathrm{\backslash nolimits}}_{i=1}^{n}\frac{1}{n}\log(x_{c,i}x_{c,i}^{T})\right) \end{array}\right.$$ (46)

Specifically, the distribution differences among EEG sample sets will be addressed by selecting the appropriate alignment centroid space through experiments.

To perform subsequent motion imagination EEG classification tasks, effective representations need to be extracted from each aligned covariance matrix. Common methods include CSP and RTS representations. Given that CSP representations are susceptible to channel selection and may not generalize well across individuals, this study chooses to extract RTS representations from the covariance matrices. Formally, RTS representations project the SPD matrix *Q*_*c,i*_ onto the tangent space of the surrounding SPD matrix *Q* to obtain Euclidean tangent space representations, computed as:


$$F_{c,i}=upper\left(\log_{Q}(Q_{ref}Q_{c,i}Q_{ref})\right)$$ (47)

where *upper*(.) denotes the extraction of the upper triangular part of the matrix to obtain Euclidean tangent space representations. The reference matrix *Q*_*ref*_=*Q*^−1/2^ is calculated from the inverse square root of the surrounding SPD matrices to ensure the homomorphism between Euclidean space representation vectors and Riemannian space SPD matrices.

### 3.3 Knowledge kernel adaptation method

After aligning samples and extracting spatial representations from each individual’s EEG data as described in the previous section, the extracted RTS spatial representations are divided into source field ${\left\{F_{s,i},y\right\}}_{i=1}^{n_{s}}$ and target field ${\left\{F_{t,i}\right\}}_{i=1}^{n_{t}}$ using M2S (multi-source to single-target) or S2S (single-source to single-target) strategies. To efficiently train a classifier in the source field that generalizes to the target field, this paper introduces a Knowledge Kernel Adaptation (KKA) method, which learns a field-agnostic kernel matrix between the two fields. Given a kernel function *k*, the KKA method constructs the kernel matrices *K*_*s*_ and *K*_*t*_ for the source and target field RTS representations, respectively, and uses these to build a field-agnostic kernel matrix *K*_*s*∪*t*_. Since the target field kernel matrix is unknown, KKA approximates the target kernel matrix *K*_*t*_ using the Nyström approximation method based on the eigen-decomposition of the known source field kernel matrix *K*_*s*_. Specifically, the KKA computation process consists of the following steps:

1) Eigen-Decomposition of the Target Kernel Matrix: First, perform eigen-decomposition on the target kernel matrix *K*_*t*_:


$$K_{t}\phi_{t}=\phi_{t}\Delta_{t}$$ (48)

where {$\phi_{t}$,Δ_*t*_} represents the eigen-system of the target kernel matrix, i.e., eigenvectors and eigenvalues. Based on Mercer’s theorem, compute the eigen-system values for the source field representation set, approximating the source field kernel matrix *K*_*s*_:


$${\tilde{\phi }}_{s}=K_{s\cup t}\phi_{t}\Delta_{t}^{-1}$$ (49)

where *K*_*s*∪*t*_ denotes the inter-individual kernel matrix, which acts as a bridge between the source and target fields and is computed using the kernel function *k*.

2) Kernel Reconstruction via Spectral Kernel Adaptation: Next, use spectral kernel adaptation [56] to reconstruct the source field kernel matrix from the approximated eigen-system. The spectral kernel design extrapolates from the target field kernel matrix’s eigen-system {$\phi_{t}$,Δ_*t*_} to obtain a generated kernel matrix for the source field representation set:


$${\tilde{K}}_{s}={\tilde{\phi }}_{s}\Delta {\tilde{\phi }}_{s}^{T}$$ (50)

where Δ represents the learning parameters, relaxed from the eigenvalues Δ_*s*_ of the source field kernel matrix. The generated source field kernel matrix retains the structural information of the eigenvectors ${\tilde{\phi }}_{s}$, and its optimal eigenvalues are obtained by minimizing the Nyström approximation error.

3) Minimizing Distribution Differences: Optimal eigenvalues Δ^*^ are used to determine the minimized distribution difference between source and target field spatial representations, making the generated kernel matrix from the source field field-agnostic. To achieve this, minimize the difference between the actual source field kernel matrix *K*_*s*_ and the generated kernel matrix${\tilde{K}}_{s}$ using the quadratic error defined as:


$$\left\{ \begin{array}{c} \underset{\Delta }{\min}{\left\|{\tilde{K}}_{s}-K_{s}\right\|}_{F}^{2}={\left\|{\tilde{\phi }}_{s}\Delta {\tilde{\phi }}_{s}^{T}-K_{s}\right\|}_{F}^{2}\\ s.t.\;\sigma_{i}\geq \mu \sigma_{i+1},i=1,2,...,m-1\\ s.t.\;\sigma_{i}\geq 0,i=1,2,...,m\\ s.t.\;\Delta =\{\sigma_{i}{\}}_{i=1}^{m}\\ s.t.\;\mu \geq 1 \end{array}\right.$$ (51)

where Δ represents *m* non-negative eigenvalues and *μ*≥1 is a damping factor that constrains the eigenvalues of the positive-definite kernel matrix to follow a power law distribution. (51) represents a typical quadratic programming problem and can be solved using MATLAB’s convex optimization toolbox (e.g., quadprog) to obtain the optimal eigenvalues Δ*.

4) Constructing the Field-Agnostic Kernel Matrix: Finally, based on the optimal source field eigenvalues Δ*, construct the approximated field-agnostic kernel matrix for both source and target fields:


$${\tilde{K}}_{s\cup t}=[\begin{array}{c} {\tilde{\phi }}_{s}\Delta^{*}{\tilde{\phi }}_{s}^{T}\\ {\tilde{\phi }}_{t}\Delta^{*}{\tilde{\phi }}_{s}^{T} \end{array}\begin{array}{c} {\tilde{\phi }}_{s}\Delta^{*}{\tilde{\phi }}_{t}^{T}\\ {\tilde{\phi }}_{t}\Delta^{*}{\tilde{\phi }}_{t}^{T} \end{array}]$$ (52)

where ${\tilde{K}}_{s\cup t}$ enables knowledge transfer between the source and target field representation sets. In the field of kernel adaptation, the most commonly used classifier is the Kernel Support Vector Machine (KSVM). Specifically, obtain the generated kernel matrix ${\tilde{K}}_{s}$ from the source field representation set using (51) and train the KSVM. Then, generalize the trained KSVM to the field-agnostic kernel matrix ${\tilde{K}}_{s}$ for predicting target field representations:


$$y_{t}={\tilde{K}}_{s\cup t}(\theta \cdot y_{s})+b$$ (53)

where *θ* and *b* represent the Lagrange multipliers and the classification margin intercept of the KSVM, respectively. The KSVM is a well-established classifier, and can be implemented using MATLAB’s libsvm toolkit, which includes adjustable penalty parameters *η*.

### 3.4 Algorithm flow

Algorithm 1 outlines the proposed FARKA method for EEG inter-individual motion imagination classification. This method includes three main components: sample alignment, RTS representation extraction, and knowledge kernel adaptation classification.

**Algorithm 1: Field-Agnostic Riemannian-Kernel Alignment Method**

Input: EEG dataset $D_{C}={\left\{x_{i},y_{i}\right\}}_{i=1}^{n}$, kernel function *k* in KKA, damping coefficient *μ*, and penalty coefficient *η* in KSVM.

Procedure:

Step 1: for *c* in (1,C):

 Compute the Riemannian space centroid of the covariance matrix for the *c*-th individual’s sample set using (42).

 Align each sample’s covariance matrix using (44).

 Extract the RTS representation vector for each sample using (49).

Step 2: for *c* in (1,C):

 Choose the representation set of the *c*-th individual as the target field and the remaining C − 1 individual representation sets as the source field (i.e., M2S); or alternatively, select another individual’s representation set as the source field (i.e., S2S).

 Compute the source field, target field, and inter-individual kernel matrices *K*_*s*_, *K*_*t*_, and *K*_*s*∪*t*_ using the kernel function *k*.

 Perform eigen-decomposition of the target kernel matrix *K*_*t*_ using (50) to obtain the eigen-system {$\phi_{t}$,Δ_*t*_}.

 Interpolate the eigen-system to the source field to obtain the interpolated eigenvectors ${\tilde{\phi }}_{s}$ using (51).

 Solve the quadratic programming problem in (53) to find the optimal eigenvalues Δ*.

 Construct the field-agnostic kernel matrix${\tilde{K}}_{s\cup t}$ based on (54), and train a KSVM classifier on the source field.

 Apply the trained KSVM classifier to obtain the classification accuracy of the target field representation set.

 Return the average classification accuracy of the target field under M2S or S2S conditions.

Output: Optimal average classification accuracy

## 4 Experiments and results

### 4.1 Dataset

**Fig 2:**
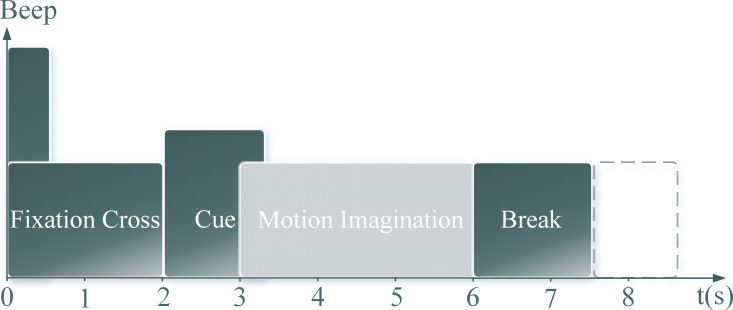
Experimental paradigm for motion imagery task acquisition. This timing diagram details the sequence of events within a single trial for the motion imagery (MI) tasks used in the public datasets. It shows the duration (in seconds, where applicable) for key phases: initial fixation/rest period, visual cue presentation indicating the MI task, the MI execution period, and the subsequent inter-trial rest interval.

To evaluate the proposed FARKA method, three commonly used BCI competition EEG datasets for MI were selected. Each dataset includes EEG samples from multiple individuals, making them suitable for testing inter-individual motion imagination EEG classification algorithms. The three chosen datasets have similar motion imagination stimulation paradigms, as shown in Fig 2.

During the experiments, individuals were seated comfortably in a chair and performed motion imagination tasks indicated by visual cues under the guidance of auditory prompts. Each sample acquisition period lasted approximately 8 seconds. Initially, there was a 2-second fixation cross to focus the individual’s attention, followed by a 1.25-second visual cue for motion imagination. The motion imagination period lasted for about 4 seconds after the appearance of the imagery prompt. After each motion imagination session, individuals had a rest period of 1.75 to 2.25 seconds before starting the next motion imagination task.

Table 1 provides statistical information about the three selected datasets. Specifically, the details of each dataset are as follows:

**Table 1:** Statistical information about the datasets.

Dataset	BCIC-IV-2a	BCIC-IV-2b	BCIC-III-4a
Number of individuals	9	9	5
Number of MI tasks	4	2	2
Sampling time points	750	750	300
Number of channels	22	3	118
Number of samples	288	400	280

1) BCI Competition IV Dataset 2a (BCIC-IV-2a) [57]: This dataset includes EEG samples from 9 healthy individuals, with 4 categories of motion imagination tasks (left hand, right hand, both feet, and tongue). The experiment consists of two acquisition periods: a training period and a testing period. We used the EEG samples from the training period to test the proposed algorithm. Specifically, each individual has 288 EEG samples, with 72 samples for each category. The EEG samples were recorded with 22 channels at a sampling rate of 250 Hz, with data from 3.5 seconds during the motion imagination period, resulting in 750 samples per channel.
2) BCI Competition IV Dataset 2b (BCIC-IV-2b) [58]: This dataset also includes EEG samples from 9 healthy individuals, with 2 categories of motion imagination tasks (left hand and right hand). The experiment consists of five acquisition periods. We used EEG samples from the first three periods to test the proposed algorithm. Each individual has 200 samples for left hand and 200 samples for right hand. The EEG samples were recorded with 3 channels at a sampling rate of 250 Hz, with data from 3.5 seconds during the motion imagination period, resulting in 750 samples per channel.
3) BCI Competition III Dataset 4a (BCIC-III-4a) [59]: This dataset includes EEG samples from 5 healthy individuals, with 2 categories of motion imagination tasks (right hand and both feet). The experiment consists of two acquisition periods. We used all EEG samples to test the proposed algorithm. Each individual has 140 samples for right hand and 140 samples for both feet. The EEG samples were recorded with 118 channels at a sampling rate of 100 Hz, with data from 3 seconds during the motion imagination period, resulting in 300 samples per channel.

### 4.2 Evaluation index and experiment settings

In the experiments, for fair comparison, the classification performance is evaluated using the classification accuracy of the target field *D*_*t*_:


$$Accuracy=\frac{\left|x:x\in D\wedge \hat{y}(x)=y(x)\right|}{\left|x:x\in D_{t}\right|}$$ (54)

where *y*(*x*) and $\hat{y}(x)$ represent the true label and predicted label of the sample in the target field, respectively. Under both M2S and S2S inter-individual motion imagination EEG classification strategies, the average classification accuracy of the entire dataset is used as the final evaluation metric.

The experiments are conducted on a hardware and software platform consisting of an Intel(R) i7-8565U CPU with 16GB of memory, running on Windows 11, and the algorithms are implemented using MATLAB 2023a. The quadratic programming problem is solved using the quadprog function, and the KSVM classifier is constructed using the libsvm toolbox.

### 4.3 Data pre-processing

For the EEG samples in each dataset, a 50th-order band-pass filter with a frequency range of 8–30 Hz was applied for preprocessing. This step removes noise and artifacts, yielding clean and effective motion imagination-related components (covering *mu* and *beta* rhythms). For the EEG sample set$D_{C}={\left\{x_{i},y_{i}\right\}}_{i=1}^{n}$ with *C* individuals, where each individual has *n* samples, each EEG sample can be represented as *x* ∈ *R*^ch×T^, where *ch* denotes the number of EEG channels, *T* represents the number of sampling points, and *y* indicates the corresponding motion imagination task category. The goal of inter-individual motion imagination EEG classification is to select the source field sample set $D_{s}={\left\{x_{i},y_{i}\right\}}_{i=1}^{n_{s}}$ and the target field sample set $D_{t}={\left\{x_{i},y_{i}\right\}}_{i=1}^{n_{t}}$, assuming that both the source and target fields share the same representation space *F*_s_ = *F*_t_ and label space *L*_s_ = *L*_t_, but have different marginal distributions *P*_s_(*x*_s_)≠*P*_t_(*x*_t_) and conditional distributions *P*_s_(*y*_s_|*x*_s_)≠*P*_t_(*y*_t_|*x*_t_). Our objective is to train a classifier supervised on the source field to achieve the lowest error loss on the target field.

In common inter-individual motion imagination EEG analysis, there are methods involving S2S and M2S transfers. To reasonably verify the effectiveness of the proposed method, we also conducted experiments using both S2S and M2S approaches. Assuming there are *C* individuals in the dataset, in the M2S mode, one individual’s sample set is randomly chosen as the target field, and the remaining *C* − 1 individuals’ sample sets are used as the source field, constructing *C* M2S inter-individual motion imagination EEG classification tasks. In the S2S mode, one individual’s sample set is randomly selected as the target field, and another individual’s sample set is selected as the source field, resulting in *C*(*C* − 1) S2S inter-individual tasks. Ultimately, the average classification performance under M2S and S2S modes is used as the result for inter-individual motion imagination EEG analysis on the dataset.

### 4.4 Contrast experiments

This study focuses on inter-individual binary classification experiments for motion imagination EEG. For the BCIC-IV-2a dataset, which includes four motion imagination tasks—left hand (L), right hand (R), feet (F), and tongue (T)—these tasks are divided into six binary classification experiments: BCIC-IV-2a-a (L/R), BCIC-IV-2a-b (L/F), BCIC-IV-2a-c (L/T), BCIC-IV-2a-d (R/F), BCIC-IV-2a-e (R/T), and BCIC-IV-2a-f (F/T). Additionally, the BCIC-IV-2b and BCIC-III-4a datasets, each containing two motion imagination classification tasks, were used to construct inter-individual binary classification experiments directly.

Tables 2 and 3 present the average classification accuracy results under the M2S and S2S strategies, respectively. A “N/A” indicates that open-source code is not available, and results were achieved for only some tasks. FARKA_R, FARKA_L, and FARKA_E represent methods using Riemannian Mean, log-Euclidean Mean, and Euclidean Mean, respectively, for sample alignment.

**Table 2:** Comparative experimental results under m2s strategy. * indicates the mdm method and # indicates the ra-mdm method.

Methods	BCIC-IV -2a-a	BCIC-IV-2a-b	BCIC-IV -2a-c	BCIC-IV -2a-d	BCIC-IV -2a-e	BCIC-IV -2a-f	BCIC-IV -2b	BCIC-III-4a	Mean
[67]	57.145	57.700	61.851	57.958	60.914	56.871	67.069	57.367	59.572
[68]	61.412	62.351	54.538	57.416	57.615	51.331	58.347	60.721	57.989
[69]	68.959	67.811	66.95	65.075	67.765	57.823	66.714	63.984	65.601
[61]	73.652	71.017	80.312	71.073	77.116	68.555	68.386	78.192	73.481
[62]*	59.592	59.889	55.208	56.095	53.837	52.648	58.139	60.664	57.094
[62]#	71.98	73.09	79.485	69.204	77.1	66.233	69.597	77.1	73.052
[54]	76.252	73.429	81.101	75.169	80.916	69.985	69.512	84.623	76.281
[22]	76.023	N/A	N/A	N/A	N/A	N/A	N/A	N/A	N/A
[70]	75.18	N/A	N/A	N/A	N/A	N/A	N/A	N/A	N/A
[71]	N/A	N/A	N/A	N/A	N/A	N/A	N/A	81.121	N/A
[60]	77.032	75.778	80.517	74.114	78.393	69.991	N/A	N/A	N/A
[23]	78.021	N/A	N/A	N/A	N/A	N/A	N/A	83.035	N/A
[61]	76.723	70.696	79.099	69.831	76.258	66.395	69.514	78.14	73.219
FARKA_R	75.955	76.571	82.155	75.354	79.91	72.065	69.94	85.482	77.214
FARKA_L	75.837	76.005	82.114	74.86	80.562	71.281	70.122	84.323	76.786
FARKA_E	75.308	73.741	82.188	70.957	79.207	71.458	68.129	83.689	75.544

**Table 3:** Comparative experimental results under s2s strategy. @ indicates the csp-lda method, % indicates the ea-csp-lda method, * indicates the mdm method, # indicates the ra-mdm method.

Methods	BCIC-IV -2a-a	BCIC-IV-2a-b	BCIC-IV -2a-c	BCIC-IV -2a-d	BCIC-IV -2a-e	BCIC-IV -2a-f	BCIC-IV -2b	BCIC-III-4a	Mean
[67]	55.251	55.361	56.597	55.288	54.987	51.973	59.906	57.546	55.917
[68]	58.259	56.521	59.038	54.287	56.617	53.355	58.94	60.653	57.281
[69]@	59.186	55.567	59.806	57.093	58.319	54.26	60.788	55.631	57.723
[69]%	64.505	61.288	70.104	59.249	65.003	58.678	67.856	67.412	64.255
[62]*	56.237	54.261	56.94	55.157	54.731	52.312	59.618	54.967	55.533
[62]#	66.657	69.857	76.684	65.426	71.876	59.666	67.187	75.146	69.048
[54]	68.792	64.024	71.368	64.963	69.869	60.183	66.14	80.32	68.124
[22]	69.063	N/A	N/A	N/A	N/A	N/A	N/A	N/A	N/A
[70]	68.757	N/A	N/A	N/A	N/A	N/A	N/A	N/A	N/A
[60]	67.414	65.012	73.232	60.863	69.523	65.752	N/A	N/A	N/A
[23]	69.127	N/A	N/A	N/A	N/A	N/A	N/A	77.111	N/A
[61]	65.966	62.54	71.502	61.073	66.144	59.114	67.93	71.152	65.783
FARKA_R	69.817	66.042	73.481	65.195	71.033	60.921	66.775	80.336	69.178
FARKA_L	69.523	65.826	74.195	65.103	71.722	61.015	66.632	79.495	69.238
FARKA_E	68.733	65.714	73.046	63.653	70.762	60.594	66.101	78.71	68.366

From the results in Table 2, it is evident that the FARKA_R method achieved the highest average classification accuracy across the three datasets under the M2S strategy. For the eight binary classification experiments, FARKA_R won five, demonstrating robust generalization performance across different motion imagination tasks and datasets. For BCIC-IV-2a-a and BCIC-IV-2a-e tasks, the classification performance of FARKA was inferior to [54]. This is primarily because [54] reduces the discrepancy between the source and target fields while preserving the source field’s class structure and the target field’s separability. Additionally, on the BCIC-IV-2a-a task, FARKA also performed worse than [60] and [23] methods. This is due to the former’s consideration of weighted spatial representations and regularization, leading to better regularization, and the latter’s enhancement of inter-individual distribution alignment through manifold weighting. The proposed FARKA method did not account for these aspects, and the current kernel functions do not provide deep characterization of the target field’s separability, resulting in inferior performance on these two tasks compared to [54].

[61] initially used the EA method to align EEG samples, followed by extracting CSP representations from the aligned samples, and then applied four common field adaptation methods to extract inter-field representations for inter-individual motion imagination EEG classification. Their experimental results indicated that the JDA method achieved the best inter-individual representation performance. Therefore, the fusion method of the above comprehensive algorithm [61] was compared with the proposed method, with results shown in Tables 2 and 3. For the eight classification tasks constructed in the experiments, [61] only surpassed the proposed method in classification performance for BCIC-IV-2a-a under the M2S strategy and BCIC-IV-2b under the S2S strategy. The average classification accuracy for the eight tasks under both M2S and S2S strategies was lower than that of the proposed method. Although CSP representations outperformed the proposed method in some inter-individual motion imagination EEG classification tasks, the average performance across all tasks was lower due to the difficulty in obtaining labels for calibration. The proposed method uses unsupervised RTS representations, eliminating the need for calibration labels, making it more suitable for online BCI systems with better plug-and-play performance. Additionally, [61] processes field adaptation and classification in two separate stages, leading to efficiency bottlenecks. Conversely, the proposed FARKA method integrates field adaptation and classification into a single stage, achieving higher efficiency, as shown in the following section.

### 4.5 Ablation experiment and contrast experiment

The results in Table 3 show that FARKA_R also achieved the highest average classification accuracy under the S2S strategy. Compared to the latest [60] and [23], FARKA_R demonstrated superior inter-individual motion imagination EEG classification performance under the S2S strategy. Due to significant accuracy improvements on the BCIC-IV-2a-a and BCIC-III-4a tasks, although FARKA_R won only one task, its average accuracy was higher than that of [62], which won four tasks. Additionally, FARKA_L achieved the second-best classification accuracy in four tasks. For BCIC-IV-2a-b and BCIC-IV-2a-c tasks, the MDM classifier [62] had a notable advantage due to the more stable representation of the covariance matrix in Riemannian space. Whether using [23,54], or the proposed FARKA method, the representations employed are projections of the Riemannian space covariance matrix onto the Euclidean tangent space. Research indicates that EEG samples exhibit spatial coupling and temporal variability, with the covariance matrix in Riemannian space better capturing subtle representation differences between motion imagination tasks. Projecting the covariance matrix onto the Euclidean tangent space may result in loss of key representations. For challenging motion imagination EEG classification tasks (e.g., BCIC-IV-2a-b and BCIC-IV-2a-c), the MDM classifier directly using the covariance matrix in Riemannian space achieved higher classification accuracy, whereas comparison methods using representations in the Euclidean tangent space suppressed effectiveness on these tasks. Overall, the FARKA method, with appropriate sample alignment mean computation, achieves competitive average classification accuracy under both M2S and S2S strategies, making it suitable for constructing inter-individual MI-based BCI systems.

For motion imagination EEG field adaptation classification problems with insufficient sample sizes, the complexity of deep neural network models increases with the number of layers, and there are currently no suitable methods to constrain model complexity, leading to common overfitting issues. Additionally, parameter optimization in deep neural networks uses gradient-based methods, which often result in numerous local optima during the iterative process, affecting robustness for motion imagination EEG field adaptation classification. To compare the classification performance of deep learning methods with the proposed method, common deep learning methods were selected, and classification performance was compared under the M2S strategy of BCIC-IV-2a dataset, as shown in Table 4. [63], [6 4], [6 5], and [66] are direct deep field adaptation methods, while ConvNet-KKA extracts pre-trained representations using deep convolutional neural networks and then performs field adaptation using the KKA method. The results in Table 4 show that neither the parameters extracted using the ConvNet model and then adapted using the proposed KKA method nor the field adaptation method [6 5] achieved better classification results on BCIC-IV-2a dataset compared to the proposed FARKA method. The ConvNet-KKA method, due to multiple iterations, learned individual-specific representations and ignored individual-agnostic representations, leading to decreased classification performance. Additionally, deep adversarial learning methods like [66] have advantages in solving multi-class motion imagination EEG field adaptation classification problems, using the softmax activation function to allow the encoder to handle multiple classes simultaneously. In contrast, the proposed traditional method is limited by the theoretical basis of kernel adaptation and currently only addresses binary motion imagination EEG field adaptation problems. Expanding it to multi-class problems will be a focus of future research.

**Table 4:** Comparative experimental results under M2S strategy on the BCIC-IV-2a dataset.

Methods	Accuracy
[63]	60.765
[64]	74.362
[65]	72.591
[66]	74.647
ConvNet-KKA	59.228
FARKA	76.790

### 4.5 Ablation experiments

#### 4.5.1 Module ablation.

To verify the feasibility and effectiveness of the proposed FARKA method, an module ablation study was conducted focusing on three aspects: sample alignment, spatial representation extraction, and inter-individual classification. Tables 5 and 6 present the results of the module ablation experiments. Specifically, RTS&KKA is used to evaluate the impact of sample alignment, RA&CSP&KKA assesses the superiority of RTS representations over CSP representations, and RA&RTS&KSVM examines the role of KKA.

**Table 5:** Ablation experimental results under M2S strategy.

Methods	RTS&KKA	RA&CSP&KKA	RA&RTS&KSVM	RA&RTS&KKA
BCIC-IV-2a-a	66.977	72.684	70.147	75.926
BCIC-IV-2a-b	59.378	70.601	74.004	76.604
BCIC-IV-2a-c	62.281	80.089	78.243	82.262
BCIC-IV-2a-d	66.101	70.994	70.041	75.339
BCIC-IV-2a-e	65.36	76.654	76.896	79.875
BCIC-IV-2a-f	57.611	65.425	66.131	71.901
BCIC-IV-2b	65.416	69.527	68.785	70.006
BCIC-III-4a	66.362	77.142	74.781	85.53
Mean	63.715	72.952	72.428	77.657

**Table 6:** Ablation experimental results under S2S strategy.

Methods	RTS&KKA	RA&CSP&KKA	RA&RTS&KSVM	RA&RTS&KKA
BCIC-IV-2a-a	64.122	65.774	64.204	69.657
BCIC-IV-2a-b	56.853	62.29	64.087	66.059
BCIC-IV-2a-c	56.75	73.418	74.039	73.467
BCIC-IV-2a-d	59.419	60.803	62.921	65.118
BCIC-IV-2a-e	57.138	68.967	68.241	70.915
BCIC-IV-2a-f	53.642	59.34	57.277	61.035
BCIC-IV-2b	62.342	67.244	67.191	66.855
BCIC-III-4a	68.212	70.917	69.77	80.3
Mean	59.734	66.187	66.062	69.104

The results in Tables 5 and 6 indicate that, compared to the three different settings in the module ablation experiments, the proposed FARKA method achieves the highest average classification performance under both the M2S and S2S strategies. Notably, in the M2S strategy, FARKA achieved the highest classification performance in all eight tasks, while in the S2S strategy, it achieved the highest performance in six tasks. From the comparison of module ablation results, it is evident that sample alignment, as a common preprocessing step, provides a significant improvement in classification performance with relatively low time complexity. Compared to CSP representations, RTS representations offer a more comprehensive representation, thus outperforming the former under both M2S and S2S strategies. KKA, which incurs some time complexity, constructs a field-agnostic kernel matrix between the source and target fields, leading to a significant performance improvement compared to using only the KSVM classifier. In summary, the module ablation study shows that all three steps in the proposed FARKA method are meaningful and contribute to significant performance improvements in inter-individual motion imagination EEG classification.

In motion imagination EEG field adaptation classification problems, effective representation of samples is crucial. Currently, CSP, Regularized CSP (RCSP) [72], RTS, and ConvNet pre-trained representations are prominent research topics. To further validate the effectiveness of RTS representations, we present a representation comparison experiment under the M2S strategy, as shown in Table 7, where BCIC-IV-2a represents the average accuracy across six binary classification tasks. Table 7 provides inter-individual motion imagination EEG classification results using KKA with CSP/RCSP/RTS representations before and after RA alignment, as well as ConvNet representations. The results demonstrate that RTS representations, after RA alignment, achieve the best average classification performance across the three datasets, indicating that these representations, guided by the KKA model, provide more stable field-agnostic characteristics and thus improve classification performance. Compared to supervised CSP/RCSP representations, the unsupervised RTS representations do not rely on the number of training samples; any newly acquired sample can directly compute tangent space representations, offering a significant advantage. ConNet pre-trained representations are obtained from models trained individually for each individual. Since individual-specific representations are more easily captured during model training, pre-trained representations often contain most individual field-specific characteristics. Directly applying these representations to the KKA model does not effectively extract field-agnostic representations, thus reducing field adaptation performance.

**Table 7:** Ablation representation experimental results under M2S strategy.

Methods	CSP&KKA	RA&CSP&KKA	RCSP&KKA	RA&RCSP&KKA	ConvNet&KKA	RTS&KKA	RA&RTS&KKA
BCIC-IV-2a	63.654	72.233	65.19	71.835	59.272	62.921	77.029
BCIC-IV-2b	65.32	69.489	65.52	69.456	N/A	65.294	69.88
BCIC-III-4a	64.591	77.924	60.791	77.972	N/A	66.285	85.439
Mean	64.587	73.166	63.813	73.173	N/A	64.842	77.372

**Fig 3:**
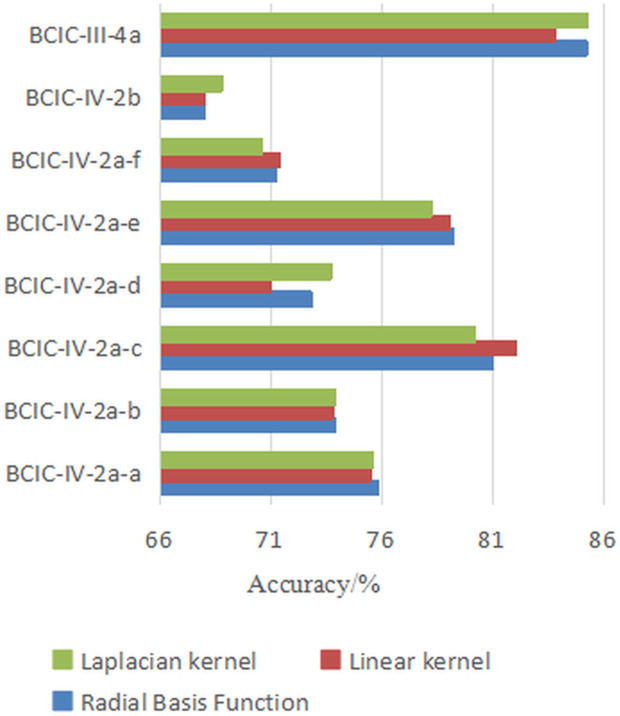
The effect of kernel function selection on performance under M2S strategy.

**Fig 4:**
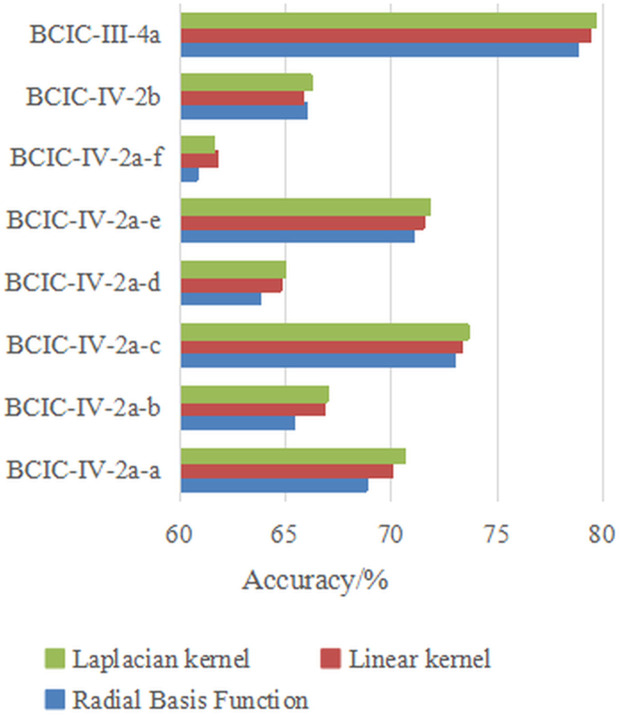
The effect of kernel function selection on performance under S2S strategy.

Additionally, the choice of kernel function *k* affects the construction of the field-agnostic kernel matrix in the FARKA method. Therefore, we used three commonly employed kernels, i.e., radial basis function $k(x,y)=\exp(-\gamma {\left\|x-y\right\|}^{2})$, linear kernel $k(x,y)=x^{T}y$, and Laplacian kernel k(x,y)=exp(−γ|x−y|) to build the FARKA method and compared the inter-individual motion imagination EEG classification performance, as shown in Figs 3 and 4. All experiments were conducted with the same damping coefficient*μ*and penalty coefficient *η*. The results indicate that for the three datasets and the eight inter-individual motion imagination EEG classification tasks, the impact of different kernel functions on the FARKA method is minimal. The method achieves good classification performance with any chosen kernel function. In practice, due to the non-linear and non-stationary nature of EEG for MI, constructing a field-agnostic kernel matrix directly can be challenging. By using RTS spatial representations, which are obtained in an unsupervised manner, we effectively decouple the spatial coupling of EEG, allowing different kernel functions to accurately capture the distinct representations of different motion imagination classes.

#### 4.5.2 Hyper-parameter ablation.

To verify the feasibility and effectiveness of the proposed FARKA method, an module ablation study was conducted focusing on three aspects: sample alignment, spatial representation extraction, and inter-individual classification. Tables 5 and 6 present the results of the module ablation experiments. Specifically, RTS&KKA is used to evaluate the impact of sample alignment, RA&CSP&KKA assesses the superiority of RTS representations over CSP representations, and RA&RTS&KSVM examines the role of KKA.

To assess the hyper-parameter impact of the proposed FARKA method, we conducted hyper-parameter ablation experiments on two hyper-parameters of the method: the damping coefficient *μ* for KKA and the penalty coefficient *η* for KSVM. Specifically, The damping coefficient *μ* in the KKA formulation (Equation 51) plays a pivotal role in regularizing the eigenvalues of the approximated kernel matrix, constraining them to follow a power-law distribution (*μ* ≥ 1). This constraint is intended to prevent overfitting to the source domain’s eigen-spectrum and promote a more generalizable kernel. The penalty coefficient *η* (often denoted as C in standard SVM formulations) in the KSVM classifier governs the trade-off between maximizing the margin and minimizing the classification error on the training data. A larger *η*imposes a higher penalty on misclassified samples.

**Fig 5:**
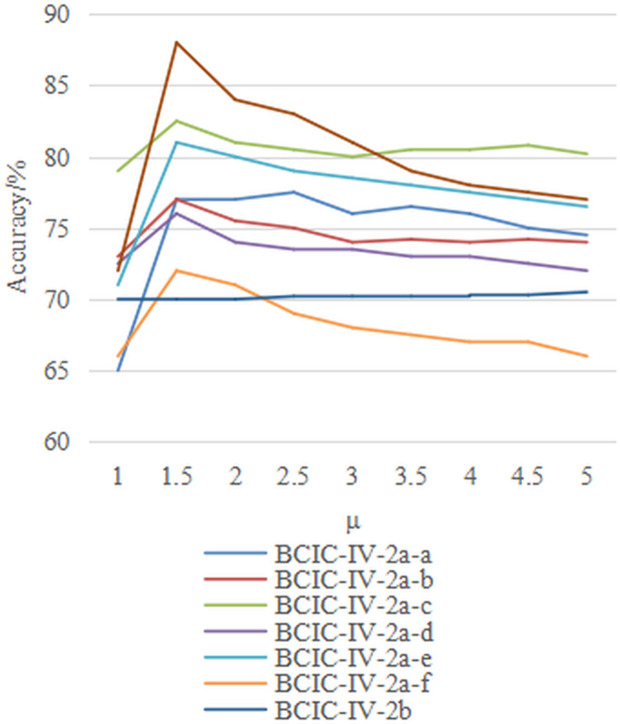
The effect of μ selection on performance under M2S strategy.

**Fig 6:**
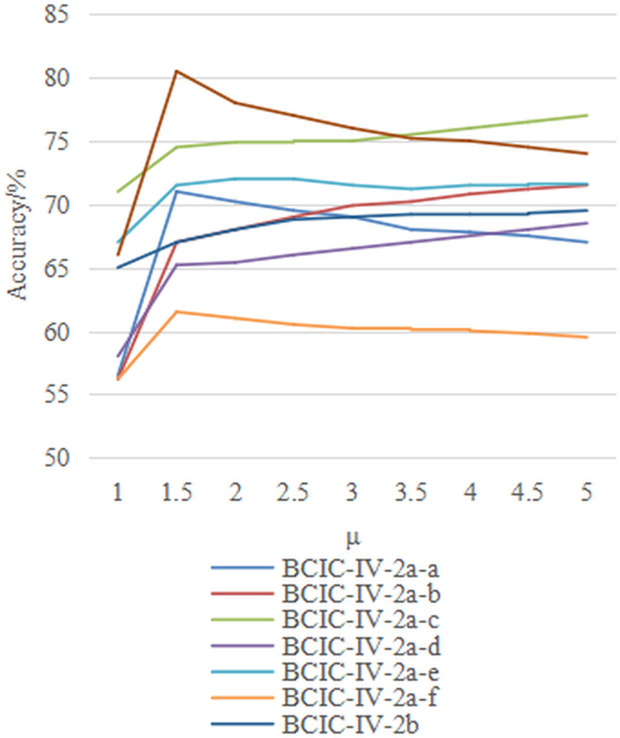
The effect of μ selection on performance under S2S strategy.

First, with the penalty coefficient *η*fixed at 10, we experimented with the damping coefficient *μ* in the range {1.0,1.5,…,5.0} across the three datasets. The experimental results under both M2S and S2S strategies are shown in Figs 5 and 6. The results indicate that across the eight inter-individual motion imagination EEG classification tasks, the damping coefficient exhibits a trend of initially increasing and then decreasing. In the M2S strategy, the optimal accuracy for all classification tasks is achieved when *μ* = 1.5. For the S2S strategy, the tasks BCIC-IV-2a-a, BCIC-III-4a, and BCIC-IV-2a-f reach their best accuracy at *μ* = 1.5, while the remaining tasks show a continuous increase in accuracy with the increase in the damping coefficient *μ*.

**Fig 7:**
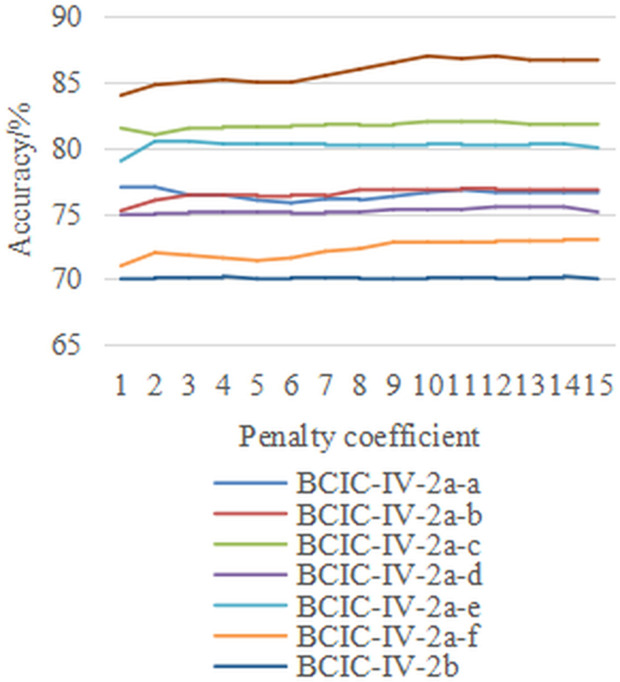
The effect of penalty coefficient selection on performance under M2S strategy.

**Fig 8:**
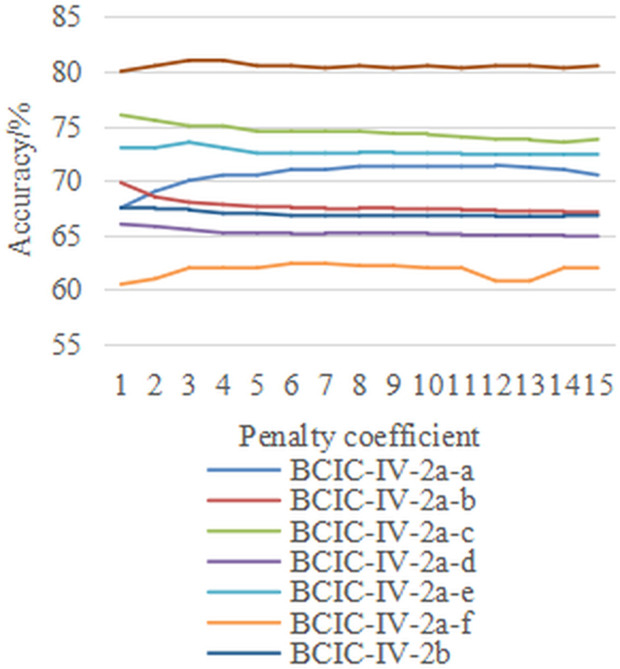
The effect of penalty coefficient selection on performance under M2S strategy.

Subsequently, with the damping coefficient *μ*fixed at 1.5, we performed experiments on the penalty coefficient *η* in the range {1,2,…,15} across the three datasets. The results under both M2S and S2S strategies are presented in Figs 7 and 8. The results show that the penalty coefficient is almost insensitive within the tested range for all eight inter-individual motion imagination EEG classification tasks. Whether under the M2S or S2S strategy, the KSVM classifiers trained using the proposed FARKA method demonstrate stable generalization performance in the target field.

### 4.6 Complexity analysis

Assuming that the source field and target field collectively contain *n* = *n*_*s*_ + *n*_*t*_ samples, we use Big-O notation to evaluate the time complexity of the proposed algorithm.

Firstly, during the sample alignment phase, computing the arithmetic mean of the covariance matrices requires *O*(*n*^2^) time complexity. Secondly, RTS representation extraction involves calculating the logarithm of each covariance matrix and extracting the upper triangular region, with a time complexity of *O*(*n·ch*^2^). Finally, for field adaptation of spatial representations using the KKA method, if the maximum dimension of the retained eigen-system is *r* and the RTS representation dimension is *ch*(*ch* − 1)/2, the total time complexity is *O*((*r* + *ch*(*ch* − 1)/2)*·n*^2^). Thus, the overall time complexity of the proposed method is *O*(*n*^2^)+*O*(*n·ch*^2^)+*O*((*r* + *ch*(*ch* − 1)/2)*·n*^2^). The numeric complexity results as shown in Tables 8 and 9.

**Table 8:** Computational complexity experiment results under M2S strategy (MS). @ indicates the  CSP-LDA method, % indicates the EA-CSP-LDA method.

Methods	[69]^@^	[69]^%^	[54]	FARKA
BCIC-IV-2a-a	652.172	771.101	869.982	661.973
BCIC-IV-2a-b	666.229	716.807	900.232	584.338
BCIC-IV-2a-c	666.878	781.739	861.92	518.6
BCIC-IV-2a-d	656.183	776.895	870.202	564.734
BCIC-IV-2a-e	657.424	764.59	861.216	642.341
BCIC-IV-2a-f	653.908	679.564	847.732	685.203
BCIC-IV-2b	491.53	506.234	1249.336	1755.476
BCIC-III-4a	488.73	490.793	1398.272	1617.26

**Table 9:** Computational complexity experiment results under S2S strategy (MS). @ indicates the CSP-LDA method, % indicates the EA-CSP-LDA method.

Methods	[69]^@^	[69]^%^	[54]	FARKA
BCIC-IV-2a-a	207.326	225.278	437.49	302.187
BCIC-IV-2a-b	213.559	237.856	429.735	299.407
BCIC-IV-2a-c	213.908	239.568	436.931	365.031
BCIC-IV-2a-d	205.675	237.622	433.103	404.965
BCIC-IV-2a-e	205.398	222.801	416.277	335.624
BCIC-IV-2a-f	209.619	232.655	410.573	331.679
BCIC-IV-2b	110.502	145.963	206.883	352.188
BCIC-III-4a	695.587	950.46	1178.249	1102.094

### 4.7 Visualizations

**Fig 9:**
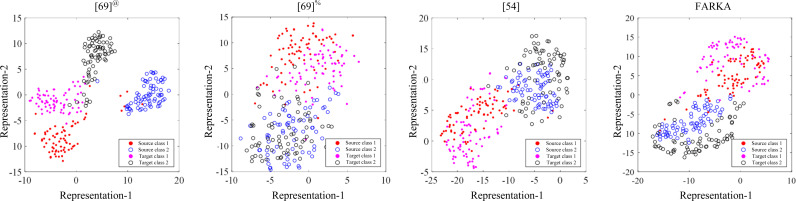
Visualizations.

To subjectively demonstrate and compare the classification performance of algorithms, we selected the BCIC-IV-2a-c classification task with Individual-2 as the source field and Individual-1 as the target field for representation distribution analysis. We compared the representation visualization of [69]^@^, [69]^%^, and [54] methods by applying the t-SNE tool to reduce the representations to 2 dimensions. Fig 9 shows the representation visualization. In the figure, source field and target field class 1 (left hand) are represented by red and purple circles, respectively, while source field and target field class 2 (feet) are represented by blue and black stars, respectively.

From the results in Fig 9, it is evident that the [69]^@^ performs the worst in distinguishing samples, with diverse sample distributions in the 2D representation space making it difficult to clearly separate the source and target fields. [69]^%^ effectively aligns the source and target field sample distributions after sample alignment; however, the sample distribution remains somewhat scattered, making it challenging to clearly differentiate between categories. [54] aligns the distributions of the source and target fields but still exhibits overlap in the boundary regions of different categories, leading to unclear classification boundaries.

In comparison to the aforementioned methods, the proposed FARKA method not only aligns the distributions of the source and target fields but also tightly clusters samples of the same category, resulting in clearer classification boundaries and higher average classification accuracy.

### 4.8 Extended evaluation and statistical analysis

To evaluate the proposed FARKA method from a statistical perspective, we conducted experiments on the OpenBMI dataset [73], which is known for its demographic diversity and is widely used as a benchmark in BCI research. The dataset includes EEG recordings from 54 adult subjects, collected using a 62-channel system with a sampling rate of 1000 Hz (typically downsampled to 100 Hz). It focuses on MI tasks, with each subject performing approximately 100 trials (50 trials for left-hand and 50 trials for right-hand tasks). The dataset’s balanced gender distribution and standardized experimental paradigm make it suitable for rigorous statistical comparisons. We compared FARKA against two state-of-the-art baseline methods: BDAN-SPD [74], which leverages motor lateralization and transformer-based spatiotemporal EEG pattern differences, and MSVTNet [75], an end-to-end multi-scale vision transformer network for MI-EEG classification.

We evaluated the methods under three scenarios: Within-Subject, Cross-Subject, and Cross-Subject with Fine-Tuning. In the Within-Subject scenario, EEG recordings from a subject’s first session were split into training and validation sets to optimize network parameters, and the trained network was evaluated on the test session data. In the Cross-Subject scenario, the network was trained on data from all other subjects and tested on the target subject’s data without prior exposure. In the Cross-Subject with Fine-Tuning scenario, the network parameters from the Cross-Subject scenario were fine-tuned using labeled EEG recordings from the target subject’s training session.

The results, as shown in Table 10, demonstrate that FARKA consistently outperforms both BDAN-SPD and MSVTNet across all scenarios. In the Within-Subject scenario, FARKA achieved an accuracy of 81.25 ± 8.45, significantly surpassing MSVTNet (74.34 ± 14.9, p < 0.01) and BDAN-SPD (79.37 ± 12.99, p < 0.05). This indicates FARKA’s robustness in handling individual EEG variations. In the Cross-Subject scenario, FARKA achieved an accuracy of 82.04 ± 9.22, outperforming BDAN-SPD (81.10 ± 12.50, p < 0.05) and MSVTNet (77.41 ± 11.53, p < 0.01), highlighting its superior generalization across subjects. In the Cross-Subject with Fine-Tuning scenario, FARKA achieved an accuracy of 83.02 ± 8.25, showing no significant difference from BDAN-SPD (83.14 ± 11.75, p > 0.05) but significantly outperforming MSVTNet (79.30 ± 11.40, p < 0.01).

**Table 10:** Comparison of accuracy(Mean ± STD) across different methods and scenarios in the openBMI dataset. the symbols * and ** indicate that the accuracy of the proposed method is statistically superior to the baseline method at significance levels of p < 0.05 and p < 0.01, respectively.

Methods	Within-Subject	Cross-Subject	Cross-Subject with Fine-Tuning
MSVTNet	74.34 ± 14.9**	77.41 ± 11.53**	79.30 ± 11.40**
BDAN-SPD	79.37 ± 12.99*	81.10 ± 12.50*	83.14 ± 11.75
FARKA	81.25 ± 8.45	82.04 ± 9.22	83.02 ± 8.25

Overall, FARKA demonstrates superior performance in handling both intra-subject and inter-subject variability, outperforming BDAN-SPD and MSVTNet in most scenarios. BDAN-SPD shows competitive performance, particularly in fine-tuning, while MSVTNet, despite its multi-scale feature extraction capabilities, lags behind in accuracy and generalization. These results underscore FARKA’s effectiveness in EEG-based motor imagery classification tasks.

### 4.9 Performance evaluation under simulated signal degradation

The real-world utility of any BCI paradigm, particularly those reliant on nuanced electrophysiological signatures like motor imagery, hinges on its resilience to suboptimal signal quality. Such degradation can stem from myriad factors, including participant fatigue, attentional lapses, or indeed, the very “low-speed” or poorly articulated imagined movements. To rigorously probe the robustness of our proposed FARKA against such eventualities, we embarked on a series of targeted simulations designed to mimic two common forms of signal compromise: diminished signal strength and increased ambient noise. These investigations were conducted on the BCIC-IV-2a dataset, specifically focusing on the binary left-hand versus right-hand MI classification (task 2a-a), employing the established M2S cross-subject validation strategy. All computational experiments leveraged the same hardware and software environment detailed previously to maintain consistency.

#### 4.9.1 Resilience to signal amplitude attenuation.

**Fig 10:**
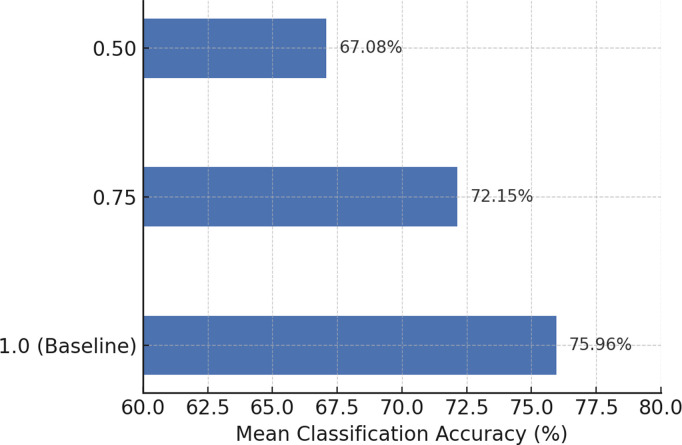
FARKA performance under signal amplitude attenuation.

One plausible manifestation of “low-speed” or weak motor imagery is a reduction in the overall amplitude of the event-related desynchronization/synchronization (ERD/ERS) phenomena, the cornerstone of MI-BCI. To simulate this, we systematically attenuated the pre-processed (8–30 Hz band-pass filtered) EEG epochs by applying a global multiplicative scaling factor (Attenuation Factor, AF) prior to feeding them into the FARKA pipeline. The baseline condition (AF = 1.0) represents the original, unattenuated signals. We then explored two levels of signal weakening: a 25% reduction in amplitude (AF = 0.75) and a more substantial 50% reduction (AF = 0.50). The impact of these manipulations on FARKA’s classification accuracy is presented in Fig 10.

From the results in Fig 10, a 25% attenuation in signal amplitude, which represents a moderate weakening of the MI-induced neural signatures, resulted in a relatively modest drop in accuracy of approximately 3.81%. This suggests that FARKA, through its Riemannian alignment and kernel adaptation mechanisms, is not inordinately reliant on a high-amplitude pristine signal and can still discern discriminative patterns even when their overt strength is diminished. More tellingly, even when the signal amplitude was halved (AF = 0.50)—a fairly severe degradation—the classification accuracy, while understandably lower at around 67.08%, did not plummet to chance levels (50% for this binary task). This persistence indicates that the underlying geometric and relational features captured by RTS and KKA retain a degree of separability even when the raw signal power is substantially curtailed.

#### 4.9.2 Tolerance to additive white gaussian noise.

**Fig 11:**
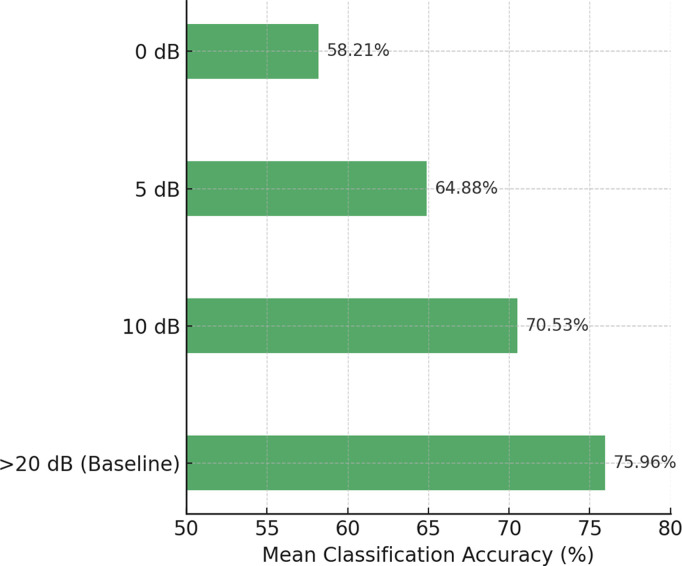
FARKA Performance under varying snr conditions.

Beyond intrinsic signal weakness, extrinsic noise contamination is an ever-present challenge in EEG recordings. To assess FARKA’s fortitude in the face of such interference, we introduced Additive White Gaussian Noise (AWGN) to the pre-processed EEG signals at varying Signal-to-Noise Ratios (SNRs). The SNR, defined as $10\times \mathrm{lg}(P_{signal}/P_{noise})$, where *P*_*signal*_ is the average power of the original filtered signal and *P*_*noise*_ is the power of the added noise, quantifies the relative strength of the signal against the noise. We evaluated performance at SNRs of 10 dB (moderate noise), 5 dB (significant noise), and 0 dB (signal and noise power are equal, a very harsh condition). The original, unadulterated signal serves as a high-SNR baseline. Fig 11 summarizes these results.

From the results in Fig 10, increasing noise levels progressively eroded classification accuracy. At an SNR of 10 dB, a condition often encountered in less-than-ideal recording environments, FARKA’s accuracy dipped by approximately 5.43%. While a noticeable decrease, the performance remains well within a usable range, suggesting that the method’s feature extraction and domain adaptation pipeline can effectively contend with a moderate degree of noise. The challenge, however, becomes more pronounced at an SNR of 5 dB, where the accuracy drop exceeded 11%. This is a scenario where the noise energy is becoming a substantial fraction of the signal energy, inevitably obscuring some of the finer discriminative details that FARKA seeks to exploit. Nevertheless, even here, the system performs considerably better than random guessing. The most stringent test, an SNR of 0 dB, pushed the accuracy down by nearly 18%. Yet, even under this extreme duress, where the signal is essentially swimming in an equivalent amount of noise, FARKA managed to eke out a performance (around 58.21%) that still holds a statistical advantage over chance. This suggests that the Riemannian manifold-based representation and the kernel learning strategy are not entirely overwhelmed, retaining some capacity to identify underlying MI patterns even when heavily masked. This resilience, particularly at 0 dB and 5 dB, is noteworthy, as it speaks to the potential of FARKA to function, albeit with reduced efficacy, in environments or with participants where EEG signal quality is a significant concern, perhaps due to “low-speed” or inconsistent mental task execution leading to signals that are easily swamped by noise. The inherent structure-preserving nature of Riemannian geometry and the robust mapping facilitated by KKA likely contribute to this ability to withstand, to a degree, the stochastic onslaught of noise.

### Ethical statement

All datasets used in this study are publicly available and have been acquired in accordance with the relevant ethical guidelines.

## 5. Discussion

The study of FARKA in MI classification presents important findings that not only contribute to the advancement of BCIs but also open the door for future research to improve the applicability and real-world performance of such systems. Several recent studies have provided insights that could significantly impact future MI classification research. Jeunet et al. [76] examined the variability in user performance in MI-BCIs, noting that while some users achieve good control, many still struggle with reliable control. They identified user profiles, including cognitive and spatial abilities, as key predictors of BCI control success. Their findings suggest that FARKA, with its field-agnostic representations, could benefit from personalized training protocols tailored to individual user profiles. This aligns with the potential for FARKA to adapt to varying user needs and improve its performance across a diverse population of users. Gupta et al. [77] explored the use of Riemannian geometry-based features for EEG classification, emphasizing the role of Neural Structured Learning (NSL) to maintain the similarity structure of EEG signals. Their study showed that NSL could improve classification accuracy with fewer training samples, which is highly relevant for FARKA. By leveraging Riemannian covariance features, FARKA might enhance its robustness in transfer learning and classification accuracy across different subjects, complementing future research on graph-based regularization and structured learning.

Looking ahead, we plan to expand FARKA’s validation to real-world experiments. This will include live BCI system deployment, where EEG data will be recorded in real-time to control assistive devices. This phase will test the adaptability and performance of FARKA in dynamic environments, emphasizing real-time data processing. This expansion into real-world environments will allow us to evaluate the method’s practicality in real-life applications.

Further, we intend to conduct cross-environment testing, subjecting FARKA to noise, signal artifacts, and other practical constraints commonly found in clinical or assistive technology settings. This testing will be crucial for demonstrating the system’s reliability in diverse environments, ensuring its readiness for deployment in real-world scenarios.

An important consideration in BCI systems is the interpretability of the model, especially for clinicians or trainers, who are key stakeholders in BCI applications. To address this, we will focus on developing user-friendly interfaces that allow clinicians to easily monitor the system’s performance and receive real-time feedback. These interfaces will provide intuitive displays of key information, enabling clinicians to make informed decisions, adjust BCI parameters, and provide guidance during rehabilitation or training sessions. By integrating these user-friendly tools, we aim to enhance the overall effectiveness of FARKA in clinical settings and improve training outcomes.

Finally, we plan to assess FARKA under real-time constraints by benchmarking it against existing methods in real-world environments. This evaluation will focus not only on computational efficiency, such as processing time and memory usage, but also on the system’s responsiveness and stability in live BCI applications. By testing FARKA in these practical scenarios, we aim to optimize it for real-time use in BCI systems, ensuring its reliability and performance under challenging conditions.

## 6. Conclusion

The proposed FARKA method significantly advances the classification accuracy and efficiency of MI tasks in BCI systems. By integrating RA for sample alignment, RTS representations, and KKA for learning field-agnostic kernel matrices, FARKA effectively addresses the challenges of nonlinear, non-stationary EEG signals and diverse inter-individual sample distributions. Experimental results on three public EEG datasets confirm that FARKA outperforms existing methods, offering robust generalization across different individuals and motion imagination tasks. This method demonstrates potential for real-time online MI-based BCI applications, enhancing the usability and performance of BCI systems for motion rehabilitation, training optimization, and motion control. Future research will focus on extending field-agnostic kernel matrix learning to incorporate spatial-spectral-temporal representations and combining the strengths of kernel learning machines with various classifiers for even higher performance and efficiency.
